# Incarcerated obturator hernia resulting in small bowel strangulation: A case report

**DOI:** 10.1097/MD.0000000000045412

**Published:** 2025-10-24

**Authors:** Xiangwei Li, Yawu Wu, Dongyun Xiao, Shaoxin Cai, Ming Yang, Zhihao Song, Teng Liu

**Affiliations:** a Department of Gastrointestinal Surgery, Huangpi District People’s Hospital Affiliated to Jianghan University, Wuhan, Hubei Province, China.

**Keywords:** case report, laparoscopy, obturator hernia, small bowel obstruction

## Abstract

**Rationale:**

Obturator hernia (OH) is a rare but serious cause of mechanical bowel obstruction that predominantly affects older women who are underweight. OH has a nonspecific presentation and high rates of strangulation and mortality, and it poses a significant diagnostic challenge. This case report highlights the importance of early recognition and demonstrates a minimally invasive surgical approach for OH with bowel necrosis.

**Patient concerns:**

A 68-year-old female with a body mass index of 19.1 kg/m² presented with a 48-hour history of abdominal pain, distension, and obstipation, accompanied by pain radiating from the left hip to the knee (Howship–Romberg sign).

**Diagnoses:**

Initial computed tomography (CT) findings without pelvic views were inconclusive. The diagnosis of an incarcerated left OH with small bowel strangulation was confirmed after 3 days via contrast-enhanced abdominopelvic CT.

**Interventions:**

A hybrid laparoscopy-assisted approach with a small laparotomy incision was performed for bowel resection and hernia repair.

**Outcomes:**

The patient recovered uneventfully and was discharged without complications. After 17 months of follow-up, the patient remained asymptomatic with normal eating and bowel habits and no abdominal complaints.

**Lessons:**

This case report emphasizes that OH must be considered in older women who are underweight, presenting with unexplained small bowel obstruction. Abdominopelvic CT imaging is the gold standard for OH diagnosis. A laparoscopy-assisted approach with a small incision is a feasible and beneficial surgical strategy for managing acute incarcerated OH even when intestinal resection is required, potentially reducing postoperative morbidity and hastening recovery.

**Key points**

- Obturator hernia is a rare but critical cause of mechanical bowel obstruction, especially in older women who are underweight, with high rates of strangulation and mortality.
- Abdominopelvic computed tomography imaging is the gold standard for preoperative diagnosis and is essential for early detection and surgical planning.
- Recognition of the Howship–Romberg sign, pain radiating from the hip to the knee, can be a key clinical clue for the early suspicion of an obturator hernia.
- A hybrid laparoscopy-assisted approach with a small laparotomy incision is a feasible and effective strategy for managing incarcerated obturator hernias, even when bowel necrosis is present.

## 1. Introduction

Obturator hernia (OH) is an exceedingly rare abdominal wall hernia, accounting for approximately 0.4% of all mechanical bowel obstructions, and is characterized by a high mortality rate due to delays in diagnosis and treatment.^[1]^ Its clinical presentation is often nonspecific and usually manifests as acute intestinal obstruction.^[2]^ A pathognomonic but inconsistently presentation is the Howship–Romberg sign: pain radiating along the medial thigh to the knee due to obturator nerve compression.^[3,4]^ Consequently, diagnosis is frequently delayed or missed, with many cases confirmed only during surgery or via computed tomography (CT) imaging. Abdominopelvic CT imaging is the modality of choice, offering high sensitivity and specificity for identifying the hernial sac within the obturator canal.

This case was reported in accordance with the Surgical CAse REport 2023 Criteria.^[5]^ We present a case of an older female with an incarcerated OH that was initially misdiagnosed, underscoring the critical importance of including OH in the differential diagnosis of small bowel obstruction in the relevant patient population. Furthermore, we describe a combined laparoscopic and open approach for managing intestinal necrosis, a scenario that presents a significant surgical challenge and is rarely documented in the literature. This case report contributes to the surgical literature by demonstrating a reproducible and minimally invasive technique for complex presentations, aiming to improve the outcomes for this high-risk patient group.

## 2. Case presentation

### 2.1. Patient’s history

A 68-year-old female patient presented to our hospital on December 18, 2023, with a 48-hour history of abdominal pain accompanied by the absence of flatus and bowel movements. The associated symptoms included nausea, non-bilious vomiting, and pain radiating from the left hip to the knee and calf. Her medical history was significant for a left femoral neck fracture sustained 11 years previously, which was treated with internal fixation using 3 screws (hardware not removed). She had a history of 2 spontaneous vaginal deliveries but no prior abdominal surgeries.

### 2.2. Physical examination and auxiliary investigations

**Figure 1.**
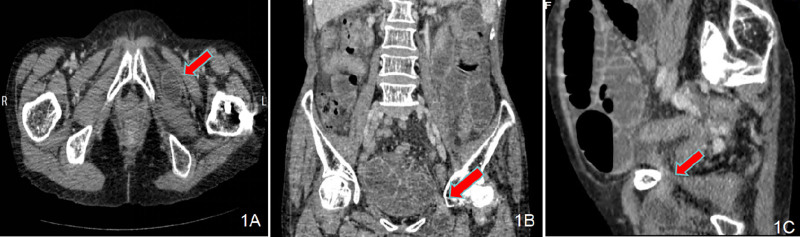
(A) Contrast-enhanced CT imaging showing the hernia sac (red arrow) between the obturator externus and the pectineus muscles passing through the left-sided obturator foramen. (B and C) A herniation of the small bowel into the left obturator canal (red arrow), with features of small intestine obstruction in the coronal and sagittal views.

Physical examination revealed the following vital signs: temperature, 36.6°C; pulse rate, 97 beats per minute; respiratory rate, 20 breaths per minute; and blood pressure, 129/84 mm Hg. Her body mass index was 19.1 kg/m². Abdominal examination revealed distension, absence of bowel sounds, a soft abdomen with tenderness in the lower quadrants, no guarding or rebound tenderness, and no palpable masses. Bowel sounds were hyperactive at 8 beats/min, and a successive splash was noted. Examination of the left hip revealed tenderness and restricted movement, whereas the left knee exhibited tenderness with a preserved range of motion and sensation. Laboratory results showed a leukocyte count of 11.69 × 10⁹/L with 77.50% neutrophils. Initial abdominal CT imaging, which did not include the pelvic floor, revealed small bowel obstruction without abnormalities in the liver, gallbladder, pancreas, spleen, or colorectum. In addition, left femoral neck fixation hardware was noted. After 3 days of conservative management (including gastrointestinal decompression, intravenous fluid therapy, antibiotics, and enemas), the patient’s abdominal distension and pain showed no improvement, and the cause of the intestinal obstruction remained undetermined. On December 21, 2023, contrast-enhanced abdominopelvic CT imaging revealed an incarcerated OH with a hernia sac located between the obturator externus and pectineus muscles (Fig. 1A). Coronal and sagittal images revealed small bowel obstruction with a small bowel loop herniating into the left obturator canal (Fig. 1B–C). Throughout the conservative treatment, the patient’s vital signs and hemodynamic status remained stable, with normal urine output.

### 2.3. Therapeutic intervention

**Figure 2.**
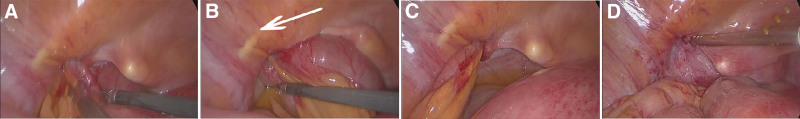
(A–C) The ileum and its mesentery are entrapped in the left obturator foramen (white arrow: Cooper ligament). (D) The herniated bowel was successfully reduced under laparoscopic guidance.

Emergency laparoscopic exploration revealed that approximately 100 cm of the ileum was proximal to the ileocecal valve, and its mesentery was entrapped within the left obturator foramen. The herniated bowel was successfully reduced under laparoscopic guidance. With laparoscopic assistance, the affected 5-cm segment of the small intestine was exteriorized, confirming necrotic changes (Fig. 2A–D). Through a 5-cm midline incision, 10 cm of the small intestine was resected, and a side-to-side anastomosis was performed and reinforced with interrupted Lambert sutures. A 50# wound protector was used for reentry into the abdominal cavity, allowing for laparoscopic repair of the OH using continuous 3-0 Prolene sutures. Postoperative management included total parenteral nutrition and antibiotics, which were initiated on postoperative day 1. Enteral nutrition was gradually introduced following the restoration of bowel function, and the patient was discharged on postoperative day 10. During hospitalization, the serum procalcitonin level decreased from 2.503 to 0.024 ng/mL, and the high-sensitivity C-reactive protein level decreased from 134.17 to <0.8 mg/L. Urine outputs on the 1st 3 postoperative days were 1150, 2000, and 1450 mL, respectively.

### 2.4. Follow-up and outcomes

**Figure 3.**
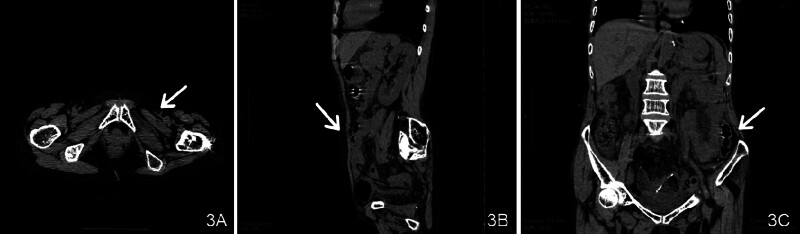
(A–C) Postoperative reexamination showing the disappearance of the hernia sac and small intestinal obstruction (white arrow).

**Figure 4.**
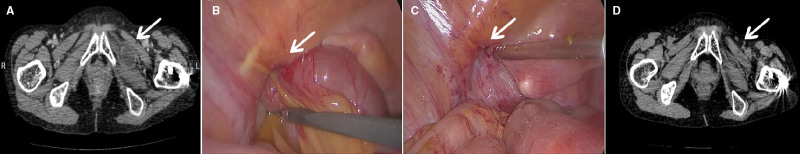
(A–D) Preoperative, intraoperative, and postoperative comparisons indicating the disappearance of the hernia sac (white arrow).

A detailed timeline is shown in Table 1, outlining the key events from admission to surgery, postoperative recovery, and follow-up. A follow-up CT scan on postoperative day 7 confirmed successful reduction of the hernial sac and resolution of the small bowel obstruction (Fig. 3A–C). Comparison of preoperative, intraoperative, and postoperative imaging findings demonstrated significant improvements in intestinal obstruction and complete resolution of the incarcerated hernia sac (Fig. 4A–D).

**Table 1:** Therapy timeline.

Preoperative	December 18 to 20, 2023	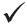 Conservative management (including gastrointestinal decompression, intravenous fluid therapy, antibiotics, and enemas).
	December 18 to 21, 2023	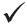 Contrast-enhanced abdominopelvic CT imaging revealed an incarcerated obturator hernia. 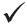 Emergency operation.
Intraoperative	December 21, 2023	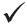 Obturator hernia with incarceration, intestinal necrosis 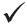 A small laparotomy incision for bowel resection and hernia repair
Postoperative	December 21, 2023 December 31, 2023	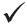 Nutritional therapy, anti-infection treatment 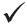 No complications, hospital discharge
Follow-up and outcomes	March 20, 2025	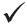 The patients recovered well 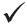 The patient remains asymptomatic with normal eating and bowel habits and no abdominal complaints.

During the patient’s hospitalization and subsequent follow-up period, no postoperative complications, such as anastomotic leakage, surgical site infection, or recurrence, were observed. After 17 months of follow-up, the patient remained asymptomatic with normal eating and bowel habits and no abdominal complaints. The patient expressed high satisfaction with the surgical outcomes and postoperative recovery process.

### 2.5. Ethical approval and informed consent

This case report did not require ethical approval from an ethics committee. Informed consent was obtained from the patient for the use of anonymized clinical, pathological, and radiological information. The patient provided written informed consent to publish medical information anonymously.

## 3. Discussion

OH is clinically rare; hence, having substantial surgical experience is challenging. The literature primarily comprises individual case reports and small case series. Since Ronsil 1st described OH in 1742, its reported prevalence is 0.07% to 1.0%, accounting for approximately 0.4% of all mechanical bowel obstructions. Most patients diagnosed with OH are aged 70 to 80 years.^[1]^ OH occurs when the abdominal contents protrude through the obturator foramen into the pelvic cavity. This type of hernia predominantly occurs in females, with incidence rates 6 to 9 times higher than those in males. This disparity may stem from anatomical differences in the female pelvis, including a broader pelvic cavity and obturator canal, an increased pelvic diameter due to multiparity, and malnutrition.^[6]^

Most patients with OH present with acute small bowel obstruction, often with concurrent strangulation.^[7]^ The Howship–Romberg sign is documented in just over half of the affected patients and is characterized by pain along the medial thigh extending to the knee and hip joints. This symptom results from the compression of the obturator nerve between the hernial sac and the obturator canal, leading to diminished or absent adductor reflexes and resultant motor dysfunction. Abdominopelvic CT imaging demonstrates high sensitivity and specificity, serving as a critical tool for diagnosing OH with small bowel obstruction, particularly in patients with atypical presentations.^[8,9]^

In our case, the patient exhibited the classic Howship–Romberg sign; however, due to her history of internal fixation for a left femoral neck fracture, the pain was erroneously attributed to secondary femoral head necrosis. Additionally, the initial omission of pelvic CT imaging and the non-specificity of OH symptoms contributed to diagnostic delay. The immediate postoperative resolution of the Howship–Romberg sign provides compelling evidence for the initial oversight of this crucial clinical sign. The clinical diagnosis and treatment model described in such articles can be served as a reference.^[10–12]^

High rates of perioperative complications and mortality associated with incarcerated OH were reported at 26.7% and 11.6%, respectively, as well as a small bowel resection rate of 40%, underscoring the urgency of surgical intervention once diagnosis is confirmed.^[1]^

A lower abdominal open surgery is considered particularly suitable for the emergent management of OH with small bowel obstruction owing to the unrestricted visual field.^[13]^ Current literature indicates that open surgical repair with primary sutures remains a prevalent method for addressing OH, albeit with a 10% recurrence rate.^[14]^ The laparoscopic transabdominal preperitoneal technique is a safe and effective emergency mesh repair method due to advantages such as reduced postoperative pain, facilitated early mobilization, shortened hospital stay, and lower postoperative morbidity rates.^[15,16]^ Although recurrence rates are low, and mesh repair does not appear to increase the risk of surgical complications, its application must be carefully considered in the context of each patient’s clinical status, comorbidities, and degree of intraoperative contamination.^[17]^ The presence of intestinal necrosis in our patient precluded the use of mesh for repair. Previous reports on laparoscopic repair of OH typically did not involve intestinal resection. However, due to the deep anatomical location of the obturator canal, cases complicated by intestinal necrosis require both bowel resection and hernia repair, traditionally necessitating a larger lower midline incision that may increase postoperative complications.^[18–20]^ In our case, we leveraged the excellent visualization and minimally invasive advantages of laparoscopy for exploration and surgical management. Using a 5-cm lower abdominal incision, we successfully performed small bowel resection, anastomosis, hernia reduction, and repair. This approach may reduce postoperative complications and shorten the length of hospital stay.^[21]^ We believe that this hybrid technique has significant potential to facilitate postoperative recovery in similar patients.

This hybrid laparoscopy-assisted approach has certain limitations. First, it requires laparoscopic expertise and open surgical skills, which may not be available at all institutions, particularly in resource-limited settings. Second, patients with significant cardiopulmonary comorbidities may not tolerate pneumoperitoneum, which is contraindicated in laparoscopy. In these cases, the traditional open approach remains safe.

Furthermore, in cases of massive bowel distension or dense adhesions, laparoscopic visualization and manipulation can be severely compromised, potentially necessitating an open procedure. Although our patient had a favorable anatomy that allowed for successful minimally invasive management, this may not be universally applicable. Finally, because this was a single-case report, the generalizability of our technique to a broader population remains uncertain. Larger prospective studies or registries are required to validate the safety, efficacy, and long-term outcomes of this hybrid approach in patients with incarcerated OH with bowel necrosis.

## 4. Conclusions

In cases of the “Little Old Lady’s Hernia” presenting with acute intestinal obstruction, the possibility of incarcerated OH should not be overlooked. Prompt surgical intervention is crucial for preventing disease progression, and abdominopelvic CT imaging plays a pivotal role in the diagnosis and management. Laparoscopic reduction and herniorrhaphy with a small incision offer potential advantages for acute incarcerated OH, even when accompanied by intestinal necrosis.

## Author contributions

**Data curation:** Shaoxin Cai, Ming Yang, Zhihao Song, Teng Liu.

**Supervision:** Yawu Wu, Dongyun Xiao.

**Writing – original draft:** Xiangwei Li.
